# ^18^F-FDG PET/CT for Risk Stratification and Prognosis of Patients with Hypermetabolic Gastrointestinal Stromal Tumors

**DOI:** 10.3390/cancers18050717

**Published:** 2026-02-24

**Authors:** Li Zhang, Yu Liu, Chunxia Qin, Huanyu Chen, Yujun Wu, Jinbo Gui, Jingwen Wang, Yong He, Xiaoli Lan, Wei Cao

**Affiliations:** 1 Department of Nuclear Medicine, Union Hospital, Tongji Medical College, Huazhong University of Science and Technology, Wuhan 430022, China; 2 Hubei Key Laboratory of Molecular Imaging, Wuhan 430022, China; 3 Department of Radiology, China Three Gorges University, Affiliated Renhe Hospital, Yichang 443000, China; 4 Department of Nuclear Medicine, Zhongnan Hospital of Wuhan University, Wuhan 430071, China

**Keywords:** gastrointestinal stromal tumors, ^18^F-FDG PET/CT, risk stratification, prognosis, SUVmax

## Abstract

**Objectives:** We aimed to evaluate the value of various PET parameters derived from ^18^F- FDG PET/CT for risk stratification and prognosis of hypermetabolic gastrointestinal stromal tumors (GISTs). **Methods:** This study included 43 patients who underwent ^18^F-FDG PET/CT imaging with hypermetabolic (SUVmax > 2.5) GIST and underwent surgical treatment. Clinicopathological characteristics, risk stratification, PET parameters including standard uptake values (SUVs), metabolic tumor volume (MTV), total lesion glycolysis (TLG), and heterogeneity index (HI), and follow-up data were reviewed. The relationship between PET parameters and risk stratification based on the modified National Institutes of Health (NIH) criteria was analyzed. PET parameters were assessed to predict relapse-free survival (RFS) and overall survival (OS), based on Cox regression analysis and Kaplan–Meier analysis. **Results:** The median follow-up duration was 50 months. During follow-up, 11 patients (25.58%) experienced recurrence and 8 (18.60%) died. In risk stratification, the high-risk group exhibited more frequent extragastric location, larger tumor size, higher mitotic count, and elevated PET parameters except SUVmax. MTV (≤32.68 vs. >32.68, 95% CI: 1.358–72.048, *p* = 0.024) emerged as an independent PET parameter of risk stratification. In univariate analysis, tumor location (gastric vs. extragastric), SUVmax (≤10.25 vs. >10.25), and HI (≤2.44 vs. >2.44) were significant prognostic factors for RFS. Tumor location and SUVmax were significant to OS on univariate analysis. However, in multivariate analysis, only SUVmax (95% CI: 1.549–46.071, *p* = 0.014) was an independent prognostic factor for both RFS and OS. **Conclusions:** ^18^F-FDG PET/CT demonstrates predictive value for hypermetabolic GIST patients. MTV derived from ^18^F-FDG PET/CT improves the ability of predicting risk stratification. SUVmax is an effective predictor of both RFS and OS.

## Simple Summary

Gastrointestinal stromal tumor (GIST) is the most common mesenchymal neoplasm of the gastrointestinal tract. Currently, risk stratification of GISTs is based on the modified National Institutes of Health (NIH) criteria. ^18^F-FDG PET/CT is increasingly used for biological risk assessment, staging, and treatment response evaluation in GISTs. Studies show that hypometabolic GISTs generally have a lower risk stratification and better prognosis. Conversely, hypermetabolic GISTs carry high risk and are associated with a poorer prognosis, thus requiring more clinical attention. To our knowledge, no study has specifically investigated risk stratification and prognosis of hypermetabolic (SUVmax > 2.5) GISTs. These results suggest that PET parameters may assist in predicting risk stratification and prognosis in GIST patients.

## 1. Introduction

GISTs represent the most prevalent mesenchymal tumors within the gastrointestinal tract, with a primary origin in the stomach and small intestine [1,2,3]. The main treatment for localized GISTs is surgical removal [4,5]. The pathogenesis of GISTs is predominantly driven by mutations in KIT (exons 11 or 9) or PDGFRA (exon 18). This specific genotype therefore serves as the cornerstone for guiding treatment with tyrosine kinase inhibitors (TKIs) like imatinib [1,6]. Aggressive GISTs have a high risk of metastasis and recurrence, making early differentiation of malignant behavior crucial [7]. Risk stratification is commonly based on the modified National Institutes of Health (NIH) criteria, which incorporate tumor size, mitotic count, primary tumor location, and rupture [8,9]. Accurate risk stratification is essential for assessing recurrence and metastasis risk post-surgery and guiding adjuvant therapy. However, preoperative assessment is challenging due to limited biopsy material and sampling variability.

Early-stage GISTs are often asymptomatic, with tumors < 2 cm typically detected incidentally [1]. Symptomatic presentations include abdominal pain, a palpable mass, or gastrointestinal bleeding [4,10]. These tumors are usually detected by imaging studies such as CT or MRI. To further evaluate the tumor’s malignant potential and metastasis status, a subset of patients may undergo ^18^F-FDG-PET/CT imaging [11]. The prognosis of GISTs varies significantly. Patients with very low-/low-risk stratification have 5-year survival > 95% after surgery. In contrast, high-risk patients face a >50% recurrence risk with surgery alone. Adjuvant TKI therapy improves 5-year relapse-free survival (RFS) to approximately 70% and 5-year overall survival (OS) to around 90%. Thus, standardized management is critical for high-risk GISTs [1,12].

With advancements in imaging, an increasing number of modalities now enable the non-invasive prediction of malignant risk and prognosis. Although, CT and MRI are the most commonly used imaging scan [13,14,15,16], ^18^F-FDG PET/CT is a crucial modality in the diagnosis of GISTs. In a cohort of 32 GIST patients utilizing ^18^F-FDG PET/CT, a significant correlation between tumor metabolic activity and clinical risk stratification was established. [17]. In research of Albano et al., metabolic tumor volume (MTV) and total lesion glycolysis (TLG) serve as independent prognostic indexes for progression-free survival in patients with GISTs [18].

^18^F-FDG PET/CT cancer imaging indicates the glucose metabolism rate of tumors, revealing malignant potential [19]. Recently, ^18^F-FDG PET/CT has been increasingly used for biological risk assessment, prognosis, and treatment response evaluation in GISTs [20,21,22,23]. The GIST patients with primary lesions of low SUVmax tend to have better prognosis [17,18]; these tumors are consequently less frequently the focus of clinical research. Although high-risk GISTs often show ^18^F-FDG avidity, some hypermetabolic GISTs are still classified as non-high based on pathology after post-resection. The risk stratification and prognosis of hypermetabolic GISTs have not been systematically reported [24]. This study was designed to evaluate the value of PET parameters in predicting risk stratification and prognosis in hypermetabolic GISTs.

## 2. Materials and Methods

### 2.1. Patients’ Enrollment

**Figure 1:**
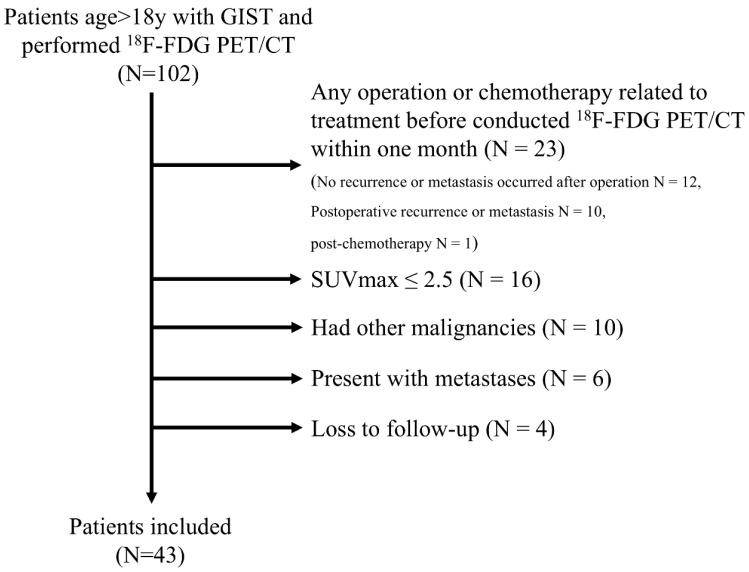
Flowchart of patient enrollment.

A total of 102 GIST patients who underwent ^18^F-FDG PET/CT scans at two Chinese medical centers between March 2010 and January 2025 were included and analyzed. Inclusion criteria were: (1) preoperative ^18^F-FDG PET/CT; (2) pathological confirmation of GIST; and (3) complete clinical data. Exclusion criteria were: (1) any operation or chemotherapy related to treatment before conducted ^18^F-FDG PET/CT; (2) hypometabolic GISTs with SUVmax ≤ 2.5 (lesions with SUVmax > 2.5 was defined as hypermetabolic GISTs); (3) existence of any malignant tumor; (4) presence of metastases; and (5) lost to follow-up. The flowchart was shown in Figure 1.

### 2.2. PET Imaging Acquisition Protocol

PET/CT scans were conducted with the CT-S64 (Siemens, Munich, Germany), as well as Discovery VCT and Discovery LS (both from GE Healthcare, Chicago, IL, USA). ^18^F-FDG (≥95% radiochemical purity) was produced using a GE Minitrace cyclotron. Prior to tracer injection, all patients were required to fast for a minimum of 6 h and to verify that their serum glucose was ≤11.1 mmol/L. An intravenous dose of 3.8–5.5 MBq/kg of ^18^F-FDG was administered, followed by an uptake period of 60 ± 5 min before image acquisition. The scanned data underwent attenuation correction and reconstruction, and were subsequently transferred to a Xeleris processing station (GE Healthcare) to generate PET, CT, and fused PET/CT images in transverse, sagittal, and coronal orientations.

### 2.3. PET Image Analysis

The PET/CT pictures were assessed independently by two seasoned specialists. Semiquantitative analysis was performed to distinguish the lesion demonstrating the highest level of ^18^F-FDG uptake. Region of interest (ROI) was delineated in the transverse plane using an SUVmax threshold of 2.5 for the calculation of MTV, TLG and heterogeneity index (HI). Intratumoral metabolic heterogeneity was quantified using the HI, defined as SUVmax/SUVmean, and it was automatically calculated by the workstation (AW4.6; GE Healthcare).

### 2.4. Follow-Up

Patients were monitored by examining the primary lesions and confirming recurrence through biopsy results, surgical findings, imaging data, or extended patient follow-up. The follow-up data cutoff was January 2025. RFS was the time from curative treatment until the cancer returns or death occurs, measuring the success of therapy in preventing relapse. OS was the time from diagnosis or treatment start until death, representing the ultimate benchmark of treatment efficacy.

### 2.5. Statistical Analysis

The distribution of quantitative variables was analyzed between groups. Quantitative data were tested for normality. Normally distributed data were shown using mean ± standard deviation or median. The data were examined using either the T-test or the Mann–Whitney U test, while categorical data were assessed with Fisher’s exact test and Pearson’s test. The best cutoff values were calculated based on Youden index for ROC curve of PET parameter. For the prediction of modified NIH consensus criteria, logistic regression was performed. Univariate and multivariate Cox analysis were performed to for RFS and OS. Kaplan–Meier approach was employed to estimate RFS and OS, while the log-rank test assessed the prognostic importance of significant factors.

All statistical analyses were performed using R 4.5.1, GraphPad PRISM 8.3 (USA) and MedCalc 19.7 (CN). We defined statistical significance as a two-sided *p* value of less than 0.05.

## 3. Results

### 3.1. Patient Clinicopathologic Characteristics

The clinicopathologic characteristics of 43 hypermetabolic GIST patients are summarized in Table 1. Based on modified NIH consensus criteria, patients were stratified in non-high-risk (n = 20) group and high-risk (n = 23) group. The median follow-up time was 50 months (18–120). In follow-up, recurrence occurred in 11 patients (25.6%), and 8 patients (18.6%) died. The median RFS and OS were not reached.

Tumors with high-risk were significantly associated with larger tumor size (>5 cm, 78.3% vs. 20.0%, *p* < 0.001), extragastric location (65.2% vs. 25.0%, *p* = 0.008), elevated mitotic counts (>5/HPF, 52.2% vs. 5.0%, *p* = 0.001), higher MTV (136.4 vs. 22.0, *p* = 0.018), TLG (704.0 vs. 154.3, *p* = 0.044), and HI (2.30 vs. 1.91, *p* = 0.03). Both RFS and OS were worse in high-risk group. No significant differences were found in gender, age, Ki-67, or SUVmax (illustrated in Table 1).

### 3.2. ROC Curve of PET Parameters for Risk Stratification

Among the 43 patients, ROC curve analysis demonstrated significant differences in PET parameters (SUVmax; MTV; TLG; HI) in relation to risk stratification (all *p* < 0.05). The optimal cutoff values for SUVmax, MTV, TLG, and HI were 7.7, 32.68 cm^3^, 122.5, and 2.25, respectively. Area under the curve (AUC) of SUVmax, MTV, TLG, and HI were 0.677 (95% CI: 0.517–0.812, *p* = 0.036), 0.822 (95% CI: 0.675–0.921, *p* < 0.001), 0.804 (95% CI: 0.655–0.909, *p* < 0.001), and 0.705 (95% CI: 0.547–0.834, *p* = 0.012). Details are in Table S1.

### 3.3. Univariate and Multivariate Analyses of Risk Stratification

A multicollinearity analysis was first conducted for all variables included in the analysis before univariate and multivariate analyses, which indicated the existence of multicollinearity between MTV and TLG due to the VIF > 10 and tolerance < 0.1 (Table S2). Therefore, we constructed a series of multivariable models. Each model contained the same core clinical covariates (e.g., tumor size, location) but included only one PET parameter at a time. Univariate analysis of preoperative clinical parameters and PET parameters showed that tumor size, tumor location, SUVmax, MTV, TLG, and HI (all *p* < 0.05) were statistically significant. In multivariate analyses, MTV (95% CI 1.358–72.048, *p* = 0.024) and tumor size (95% CI 1.10–53.343, *p* = 0.04) were independent predictors of risk stratification (Table 2).

### 3.4. ROC Curve of ^18^F-FDG PET/CT Metabolic Parameters for Prognosis

The cutoff value was set as 10.25 for SUVmax, 6.65 cm^3^ for MTV, 207.41 for TLG, and 2.44 for HI. The AUC of SUVmax for RFS was 0.710 (95% CI: 0.552–0.838, *p* = 0.0125), and that of OS was 0.723 (95% CI: 0.566–0.849, *p* = 0.035), respectively. The AUC of MTV for RFS was 0.550 (95% CI: 0.391–0.702, *p* = 0.497), and that of OS was 0.65 (95% CI: 0.490–0.789, *p* = 0.171), respectively. The AUC of TLG for RFS was 0.646 (95% CI: 0.486–0.786, *p* = 0.093), and that of OS was 0.689 (95% CI: 0.530–0.821, *p* = 0.0878), respectively. The AUC of HI for RFS was 0.706 (95% CI: 0.547–0.835, *p* = 0.006), and that of OS was 0.707 (95% CI: 0.549–0.836, *p* = 0.0485), respectively (Table S3).

### 3.5. Univariate and Multivariate Analyses for RFS

The median follow-up was 50 months (ranging from 18 to 120 months). Eleven patients experienced a recurrence, averaging 37 months. The median RFS remains unreached. In univariate analysis, tumor location (95% CI 1.574–46.884, *p* = 0.013), SUVmax (95% CI 1.725–40.907, *p* = 0.008), and HI (95% CI 1.217–35.529, *p* = 0.029) showed significantly for RFS. In multivariate analysis, tumor location (95% CI 1.156–41.763, *p* = 0.034), and SUVmax (95% CI 1.177–36.735, *p* = 0.032) were confirmed to be independent prognostic factors for RFS (Table 3).

**Figure 2:**
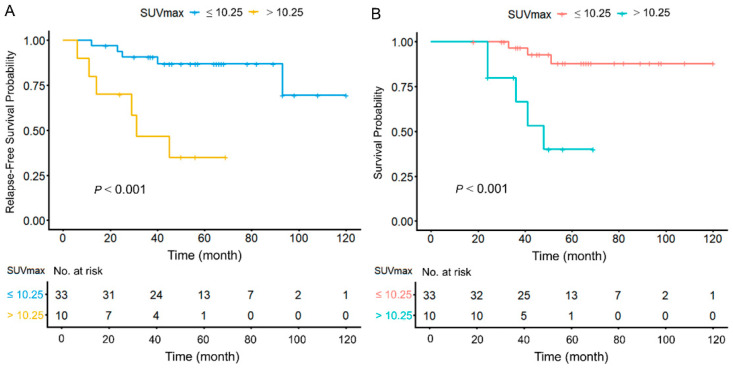
Kaplan–Meier curves for (**A**) relapse-free survival (RFS) and (**B**) overall survival (OS) in patients with GISTs.

In Kaplan–Meier analysis, the median RFS for patients with SUVmax > 10.25 was 31 months. However, patients with SUVmax ≤ 10.25 did not reach the median RFS (95% CI 89.551–116.456). Patients with SUVmax > 10.25 had shorter RFS (95% CI 77.985–106.215, *p* < 0.001, Figure 2A).

### 3.6. Univariate and Multivariate Analyses for OS

For OS, the median OS has not been reached. Eight patients had a mean survival time of 30 months. In univariate analysis, tumor location (95% CI, 1.124–74.346, *p* = 0.039) and SUVmax (95% CI, 2.619–70.673, *p* = 0.005) showed a significant correlation with OS. Other preoperative parameters such as tumor size, MTV, TLG, and HI did not have significant prognostic value. In multivariate analysis, SUVmax (95% CI 1.549–46.071, *p* = 0.014) was confirmed to be an independent prognostic factor for OS (Table 4).

**Figure 3:**
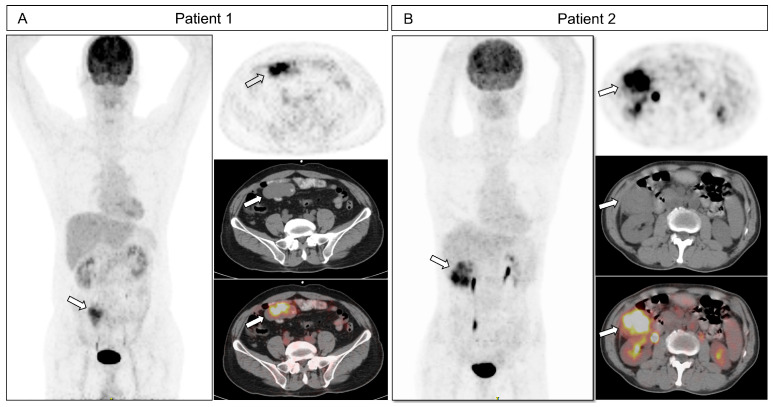
Representative cases. (**A**) A 67-year-old male diagnosed with a small intestinal GIST (size 6.4 cm, high-risk NIH criteria, white arrow). The SUVmax was 8.5, MTV was 43.52 cm^3^, and TLG was 177.13. The patient overall survival was noted at 67 months after surgery without recurrence. (**B**) A 69-year-old man with small intestinal GIST (size 6.3 cm, high-risk NIH criteria, white arrow). The SUVmax was 11.37, MTV was 130.0 cm^3^, and TLG was 601.9. The patient died and the overall survival was noted at 24 months after surgery.

In Kaplan–Meier analysis, patients with SUVmax > 10.25 had a median OS of 48 months, while the OS of GISTs with SUVmax < 10.25 was not reached (95% CI 100.710–120.640). Patients with higher tumor size and SUVmax > 10.25 had shorter OS (95% CI 89.394–112.709, *p* < 0.001, Figure 2B). Representative ^18^F-FDG PET/CT images are shown in Figure 3. A case with a favorable prognosis (Figure 3A) involved a 6.4 cm small intestinal GIST classified as high- risk stratification, with an SUVmax of 8.5. Conversely, a case with a poor prognosis (Figure 3B) also presented with a high-risk stratification, 6.3 cm small intestinal GIST, but exhibited a higher SUVmax of 11.37 (representative cases are illustrated in Figure 3).

## 4. Discussion

Our study investigated the utility of PET parameters in predicting risk stratification and prognosis for hypermetabolic GISTs, which are considered more aggressive and associated with poorer prognoses, warranting significant attention. This may be the first study specially focusing on hypermetabolic GISTs in ^18^F-FDG PET/CT. These results demonstrated that PET parameter MTV was an independent risk factor for risk stratification, while the SUVmax emerged as independent prognostic indictor. This study highlights the potential of PET parameters in guiding clinical decision-making and improving prognostic assessment for patients with hypermetabolic GISTs.

The modified NIH consensus criteria are main evaluation framework for recurrence risk of GISTs [8]. Accurate discernment of high risk versus non-high risk is crucial for GISTs’ optimal treatment strategies [12]. However, a key limitation of the risk stratification is its reliance on postoperative pathological examination, which precludes early assessment of the malignant potential of GISTs. In addition to the modified NIH consensus criteria, the recurrence risk of GISTs is also correlated with the Ki-67 index [25,26,27], mitotic count [9,28,29], and the presence of hemorrhage or necrosis [30]. At the molecular level, the pathogenesis of GISTs is primarily due to oncogenic mutations in the KIT or PDGFRA genes, which account for approximately 80–85% of all cases. Notably, exon 11 mutations, although considered an independent unfavorable prognostic factor, exhibit high sensitivity to imatinib therapy. Conversely, exon 9 mutations are associated with a higher risk and more aggressive disease phenotype. Most non-D842V PDGFRA mutations also demonstrate a favorable response to imatinib and are associated with a relatively positive prognosis [2]. Therefore, the prognosis of GISTs are influenced by risk stratification, clinicopathological and molecular features. However, surgical intervention or biopsy procedures still carry a low risk of tumor rupture [30]. Additionally, the uneven distribution of mitotic counts within the tumor can introduce sampling bias, which may compromise the accuracy of risk stratification and prognosis predictions. These limitations underscore the need for more reliable preoperative assessment tools to guide clinical decision-making.

^18^F-FDG PET/CT visualizes glucose metabolism at a cellular level, which correlates strongly with mitotic activity and proliferation. This whole-body imaging technique functions by detecting the uptake of radiolabeled glucose analogs [31,32]. In GIST patients, the metabolic activity of lesions on PET parameters is significantly correlated with Ki-67 index, mitotic counts [33], and the presence of hemorrhage or necrosis [30]. Kazuo et al. demonstrated that tumor size, mitotic count and Ki-67 index were positively correlated with SUVmax, and further established that SUVmax can effectively identify the risk stratification of GISTs [20]. PET parameters are also significant factors influencing both risk stratification and prognosis in GISTs [17,18,24,34]. Research indicates that hypometabolic GISTs normally have lower risk stratification and better prognosis [17]. In a study by Hwang et al., patients with GIST who had SUVmax < 7.04 showed a 5-year RFS of 96.4% [24]. Another study showed that in GIST patients with SUVmax ≤ 4.2, about 60 months of both PFS and OS were 100% [18]. Therefore, hypometabolic GISTs have a low malignant risk and a favorable prognosis. Conversely, hypermetabolic GISTs carry a high risk and are associated with a poorer prognosis, thus requiring more clinical attention. To address this variability, we investigated PET parameters to distinguish the risk stratification and prognosis of patients with hypermetabolic GISTs before operation.

As the most commonly employed semi-quantitative parameter in ^18^F-FDG PET/CT, SUVmax as well as MTV and TLG is the standard for evaluating therapeutic efficacy in clinical practice [35]. HI, a new parameter reflecting intratumoral-metabolic heterogeneity which mentioned in our article before [36]. SUVmax > 2.5 is widely used as the threshold for defining metabolically active lesions [23]. All the patients with SUVmax ≤ 2.5 are non-high-risk stratification, which means they turn out to have better prognostics. Hypermetabolic GISTs, specifically, have rarely been studied. In this research, only hypermetabolic GISTs were included to be analyzed. The results indicated that MTV was an independent factor in risk stratification, and SUVmax was an independent prognostic factor. Among RFS and OS, the high-SUVmax group had shorter RFS and OS. Our research aligned with findings by Kazuo et al., who also reported a correlation between SUVmax and OS, identifying SUVmax = 5.68 as the most predictive threshold for OS in GISTs [20]. The difference in SUVmax thresholds between studies may be attributed to variations in sample size, follow-up duration, and the exclusion of patients with SUVmax values below 2.5 in our study.

This research, which has integrated an assessment of preoperative factors, offers valuable insights for risk stratification and prognosis in hypermetabolic GISTs. However, the research still has several limitations. First, the retrospective design of this study is inherently susceptible to selection bias. The small size of cases was limited. Therefore, larger number of multicenter cases should be included in the study. Second, the stomach and small intestine accounted for the majority of primary tumor sites, where physiological ^18^F-FDG uptake can occur, potentially affecting the accuracy of metabolic parameter measurements. Finally, although all ^18^F-FDG PET/CT systems underwent regular quality control, system errors are inevitable and may have influenced the results.

## 5. Conclusions

In conclusion, this study represents the inaugural investigation concentrating on the hypermetabolic GIST population in the context of risk stratification and prognosis. These results suggest that metabolic parameters derived from ^18^F-FDG PET/CT can effectively predict risk stratification and prognostic. MTV independently predicts high-risk classification. Additionally, patients with higher SUVmax were found to be associated with shorter RFS and OS. These results underscore the potential of ^18^F-FDG PET/CT as a valuable tool for guiding clinical decision-making and improving prognostic assessment in hypermetabolic GISTs.

## Figures and Tables

**Table 1:** Clinicopathologic characteristics of GIST patients.

Characteristic	All Patients (n = 43) N (%) or Average (± SD)	Risk Classifcation		*p*
		Non-High-Risk (n = 20)	High-Risk (n = 23)	
Gender				0.476
Male	33 (76.7%)	14 (70.0%)	19 (82.4%)	
Female	10 (23.3%)	6 (30.0%)	4 (17.4%)	
Age				0.639
≤60	21 (48.8%)	9 (45.0%)	12 (52.2%)	
>60	22 (51.2%)	11 (55.0%)	11 (47.8%)	
Tumor size				**<0.001**
≤5 cm	21 (48.8%)	16 (80.0%)	5 (21.7%)	
>5 cm	22 (51.2%)	4 (20.0%)	18 (78.3%)	
Tumor location				**0.008**
Gastric	23 (53.5%)	15 (75.0%)	8 (34.8%)	
Extragastric	20 (46.5%)	5 (25.0%)	15 (65.2%)	
Small bowel	16 (37.2%)	4 (20.0%)	12 (52.2%)	
Colorectum	1 (2.3%)	0 (0%)	1 (4.3%)	
Esophagus	3 (7.0%)	1 (5.0%)	2 (8.7%)	
Mitotic count				**0.001**
≤5/HPF	30 (70.0%)	19 (95.0%)	11 (47.8%)	
>5/HPF	13 (30.0%)	1 (5.0%)	12 (52.2%)	
Ki-67 score				1.422
≤5%	26 (60.5%)	14 (70.0%)	12 (52.2%)	
>5%	17 (39.5%)	6 (30.0%)	11 (47.8%)	
Hemorrhagic/necrosis				**0.004**
no	26 (60.5%)	17 (85%)	9 (39.1%)	
yes	17 (39.5%)	3 (15%)	14 (60.9%)	
SUVmax	9.9 ± 6.9	8.5 ± 7.1	11.1 ± 6.6	0.214
MTV	83.2 ± 166.5	22.0 ± 39.3	136.4 ± 212.6	**0.018**
TLG	448.3 ± 931.0	154.3 ± 445.0	704.0 ± 1156.2	**0.044**
HI	2.12 ± 0.61	1.91 ± 0.55	2.30 ± 0.60	**0.030**
RFS				**0.039**
Yes	11 (25.6%)	2 (10%)	9 (39.1%)	
No	32 (74.4%)	18 (90%)	14 (60.9%)	
OS				
Death	8 (18.6%)	1 (5.0%)	7 (30.4%)	**0.050**
Live	35 (81.4%)	19 (95.0%)	16 (69.6%)	

HPF = high-power fields; SUV = standard uptake value; MTV = metabolic tumor volume; TLG = total lesion glycolysis; HI = heterogeneity index; RFS = relapse-free survival; OS = overall survival.

**Table 2:** Univariate and multivariate Cox regression analysis for risk stratification.

Factors	Univariate Analysis		Multivariate Analysis							
	*p*	OR (95% CI)	*p*	OR (95% CI)	*p*	OR (95% CI)	*p*	OR (95% CI)	*p*	OR (95% CI)
Tumor location	**0.011**	5.625 (1.492–21.203)	0.068	5.838 (0.880–38.727)	0.057	6.273 (0.944–41.682)	0.053	6.626 (1.034–42.465)	0.055	7.691 (1.297–45.62)
Tumor size	**<0.001**	14.40 (3.287–63.082)	**0.001**	17.99 (3.076–105.263)	**0.040**	7.657 (1.099–53.343)	**0.010**	11.465 (1.801–72.967)	**0.004**	13.97 (2.319–84.149)
SUVmax	**0.027**	4.245 (1.183–15.236)	0.415	2.069 (0.361–11.871)						
MTV	**<0.001**	25.50 (4.511–144.147)			**0.024**	9.892 (1.358–72.048)				
TLG	**0.001**	20.57 (3.722–113.703)					0.064	5.559 (0.906–34.090)		
HI	**0.024**	6.923 (1.294–37.051)							0.279	3.139 (0.396–24.844)

OR = Odds ratio; CI = confidence interval; SUV = standard uptake value; MTV = metabolic tumor volume; TLG = total lesion glycolysis; HI = heterogeneity index.

**Table 3:** Univariate and multivariate Cox regression analysis for RFS.

Factors	Univariate Analysis		Multivariate Analysis			
	*p*	HR (95% CI)	*p*	HR (95% CI)	*p*	HR (95% CI)
Tumor location	**0.013**	8.591 (1.574–46.884)	**0.034**	6.948 (1.156–41.763)	0.063	5.491 (0.911–33.106)
Tumor size	0.107	3.429 (0.765–15.358)				
SUVmax	**0.008**	8.40 (1.725–40.907)	**0.032**	6.575 (1.177–36.735)		
MTV	0.518	1.761 (0.317–9.785)				
TLG	0.098	3.341 (0.801–13.943)				
HI	**0.029**	6.577 (1.217–35.529)			0.167	3.610 (0.584–22.326)

HR = hazard ratio; CI = confidence interval; SUV = standard uptake value; MTV = metabolic tumor volume; TLG = total lesion glycolysis; HI = heterogeneity index.

**Table 4:** Univariate and multivariate Cox regression analysis for OS.

Factors	Univariate Analysis			Multivariate Analysis		
	*p*	HR	95% CI	*p*	HR	95% CI
Tumor size	0.451	1.735	0.414–7.267			
Tumor location	**0.039**	9.141	1.124–74.346	0.139	5.122	0.587–44.689
SUVmax	**0.005**	13.604	2.619–70.673	**0.014**	8.447	1.549–46.071
MTV	0.416	2.740	0.332–22.613			
TLG	0.251	2.317	0.553–9.703			
HI	0.136	3.38	0.681–16.768			

HR = hazard ratio; CI = confidence interval; SUV = standard uptake value; MTV = metabolic tumor volume; TLG = total lesion glycolysis; HI = heterogeneity index.

## Data Availability

The data presented in this study are available in this article (and Supplementary Materials).

## Supplementary Materials

The following supporting information can be downloaded at https://www.mdpi.com/article/10.3390/cancers18050717/s1, Table S1: AUC of PET parameters for risk stratification, Table S2: Multicollinearity analysis for variable, Table S3: AUC of PET parameters for prognosis.

## Abbreviations

The following abbreviations are used in this manuscript:

GISTs	gastrointestinal stromal tumors
NIH	National Institutes of Health
SUV	standard uptake values
MTV	metabolic tumor volume
HI	heterogeneity index
RFS	relapse-free survival
OS	overall survival
(TKIs)	tyrosine kinase inhibitors
ROI	region of interest
