# Supplementary material for: 18F-FDG PET/CT for Risk Stratification and Prognosis of Patients with Hypermetabolic Gastrointestinal Stromal Tumors
Source: Cancers (Basel). 2026 Feb 24;18(5):717. doi: 10.3390/cancers18050717 (PMC12984645; doi:10.3390/cancers18050717)
Supplement: Supplementary file 1 [file cancers-18-00717-s001.zip › cancers-4111902-supplementary.pdf]

Table S1. AUC of PET parameters for risk stratification.

Variable	AUC	Cut-Off Value	*p*
SUVmax	0.677	7.7	**0.036**
MTV	0.822	32.68	**<0.001**
TLG	0.804	122.5	**<0.001**
HI	0.705	2.25	**0.012**

AUC = area under the curves; SUVs = standard uptake values; MTV = metabolic tumor volume; TLG = total lesion glycolysis; HI = heterogeneity index.

Table S2. Multicollinearity analysis for variable

Variable	TOL	VIF
Tumor size	0.480	2.081
SUVmax	0.126	7.907
MTV	**0.017**	**58.815**
TLG	**0.015**	**64.663**
HI	0.199	5.019

VIF = variance inflation factor; TOL = Tolerance; SUVs = standard uptake values; MTV = metabolic tumor volume; TLG = total lesion glycolysis; HI = heterogeneity index. VIF > 10 and/or TOL < 0.1 indicates serious collinearity.

Table S3. AUC of PET parameters for prognosis.

Variable	Cut-off	RFS			OS		
	Value	AUC	p	95% CI	AUC	p	95% CI
SUVmax	10.25	0.710	0.0125 *	0.552–0.838	0.723	0.035 *	0.566–0.849
MTV	6.65	0.550	0.497	0.391–0.702	0.650	0.171	0.490–0.789
TLG	207.41	0.646	0.093	0.486–0.786	0.689	0.0878	0.530–0.821
HI	2.44	0.706	0.006 **	0.547–0.835	0.707	0.0485 *	0.549–0.836

AUC = area under the curves; RFS = Relapse-free survival; OS = Overall survival; SUVs = standard uptake values; MTV = metabolic tumor volume; TLG = total lesion glycolysis; HI = heterogeneity index.
